# Supplementary material for: Automated bone marrow cytology using deep learning to generate a histogram of cell types
Source: Commun Med (Lond). 2022 Apr 20;2:45. doi: 10.1038/s43856-022-00107-6 (PMC9053230; doi:10.1038/s43856-022-00107-6)
Supplement: Supplementary file 2 — Description of Additional Supplementary Files [file 43856_2022_107_MOESM2_ESM.pdf]

Description of Additional Supplementary Files

File Name: Supplementary Data 1

Description: Source data
