# Supplementary material for: Automated bone marrow cytology using deep learning to generate a histogram of cell types
Source: Commun Med (Lond). 2022 Apr 20;2:45. doi: 10.1038/s43856-022-00107-6 (PMC9053230; doi:10.1038/s43856-022-00107-6)
Supplement: Supplementary file 3 — Supplementary information [file 43856_2022_107_MOESM3_ESM.pdf]

Supplementary Information

Tiles	Number of annotated tiles in dataset	Number of annotated and augmented tiles in the training set
Inappropriate Tiles	70250	70250
Appropriate Tiles	4750	28500

Table S1: **The number of annotated and augmented tiles in our prepared dataset from 250 individual patients' WSIs to use in training of the proposed ROI detection model.**

Object class	Number of annotated objects in dataset	Number of annotated and augmented objects in the training set
Neutrophil	2714	119416
Metamyelocyte	1017	44748
Myelocyte	1199	52756
Promyelocyte	409	17996
Blast	3950	173800
Erythroblast	2668	117392
Megakaryocyte nucleus	23	1012
Lymphocyte	1305	57420
Monocyte	569	25036
Plasma cell	176	7744
Eosinophil	249	10956
Basophil	7	308
Megakaryocyte	106	4664
Debris	5603	246532
Histiocyte	191	8404
Mast cell	33	1452
Platelet	3971	174724
Platelet clump	585	25740
Other cell	2007	88308
**Total cell annotated**	**26782**	**1178408**

Table S2: **The number of annotated and augmented cellular (white blood cells) and non-cellular objects classes in our prepared dataset belong to 500 individual patients' WSIs to use in training of the proposed cell detection and classification model.**

Diagnostic tags	Used in Training	Used in Test-Validation	Extra annotated data used in Test-Validation	Number of patients
Normal	16	4	12	32
Myelodysplastic syndrome (MDS)	10	2	12	24
Acute leukemia	10	4	3	17
Lymphoproliferative disorder	8	3	N/A	11
Plasma cell neoplasm	7	5	N/A	12
Hypercellular	1	1	N/A	2
Erythroid hyperplasia	1	0	N/A	1
Myeloproliferative neoplasm (MPN)	2	1	N/A	3
Inadequate	2	1	N/A	3
Hypocellular	1	0	N/A	1
Total	58	21	N/A	106

Table S3: **Diagnostic tags and the number of patient WSI used for training and test-validation in each category for the cell detection and classification model. The extra annotated data column, shows the number of new cases that annotated and assessed by the expert hematopathologists to evaluate normal, MDS and acute leukemia cases.**

Cell Type	AP. I1	AP. I2	AP. I3	AP. I4	AP. I5	AP. I6	AP. I7	AP. I8
Neutrophil	0.72	0.78	0.83	0.86	0.87	0.90	0.92	0.90
Metamyelocyte	0.56	0.63	0.70	0.73	0.74	0.74	0.75	0.77
Myelocyte	0.53	0.54	0.58	0.61	0.69	0.76	0.80	0.80
Promyelocyte	0.41	0.45	0.56	0.55	0.60	0.60	0.62	0.62
Blast	0.63	0.71	0.79	0.80	0.80	0.82	0.82	0.84
Erythroblast	0.69	0.75	0.78	0.84	0.87	0.91	0.91	0.92
Megakaryocyte nucleus	0.28	0.36	0.48	0.57	0.56	0.59	0.60	0.60
Lymphocyte	0.45	0.47	0.51	0.52	0.54	0.61	0.64	0.66
Monocyte	0.48	0.53	0.56	0.63	0.65	0.65	0.69	0.72
Plasma cell	0.55	0.57	0.62	0.66	0.68	0.70	0.71	0.72
Eosinophil	0.57	0.62	0.79	0.85	0.89	0.90	0.94	0.97
Megakaryocyte	0.46	0.50	0.75	0.79	0.79	0.79	0.80	0.82
Debris	0.54	0.61	0.62	0.70	0.73	0.78	0.77	0.79
Histiocyte	0.30	0.39	0.44	0.50	0.51	0.52	0.52	0.54
Platelet	0.39	0.43	0.47	0.53	0.60	0.64	0.66	0.64
Platelet clump	0.38	0.39	0.42	0.53	0.59	0.57	0.62	0.62
**Average**	**0.50**	**0.55**	**0.62**	**0.67**	**0.69**	**0.72**	**0.74**	**0.75**

Table S4: **Performance result of applying active learning by evaluating on one single validation dataset for all iterations.**

Figure S1: **The LableImg annotation interface.**

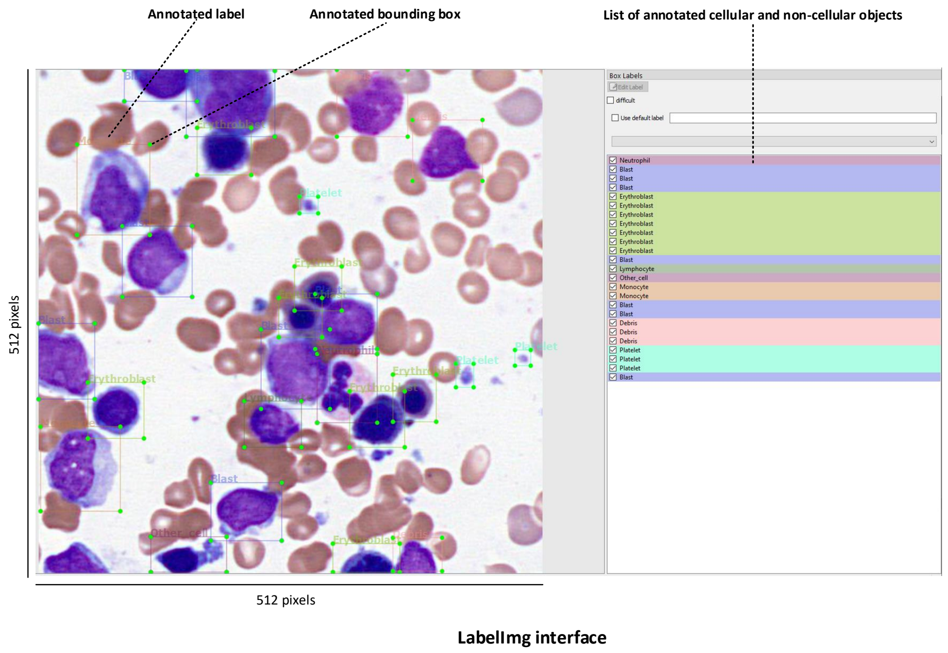

Objects within the selected tiles were annotated in both types and locations. For the types, all cellular (white blood cell) and non-cellular objects annotated by selecting the predefined object types and also localized by creating the bounding boxes to delineate the object boundary. Both these annotations have been used for training cell detection and classification model.

Figure S2: **Two samples of applying the proposed ROI detection method.**

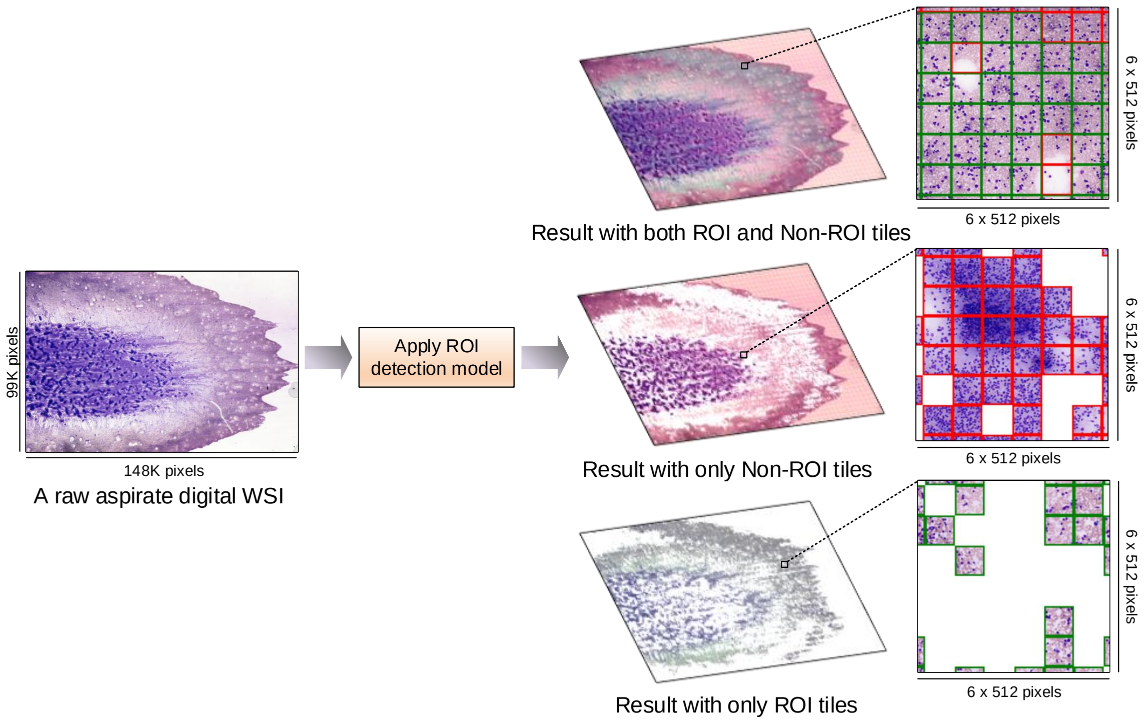

(a)

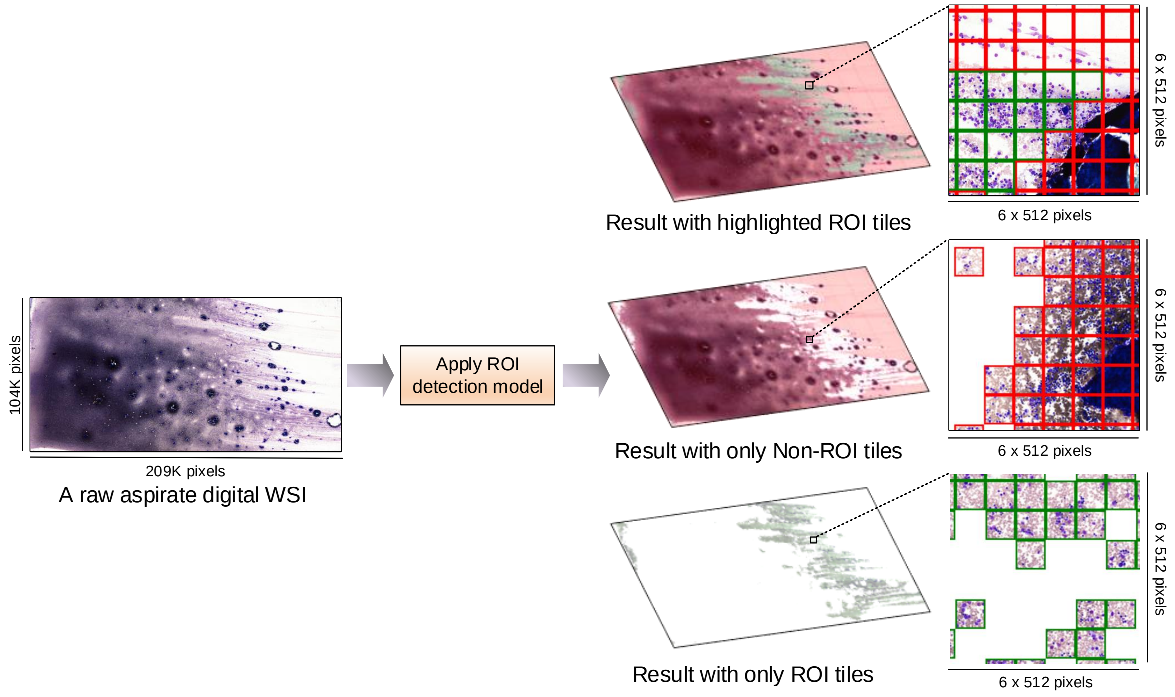

(b)

(a) A sample of applying the proposed region of interest (ROI) detection method on crush (squash) preparation aspirate specimen. (b) A sample of applying the proposed ROI detection method on push preparation aspirate specimen.

Figure S3: **Examples of annotated tiles, as region of interest (ROI) and non-region of interest (Non-ROI) for training the proposed ROI detection model.**

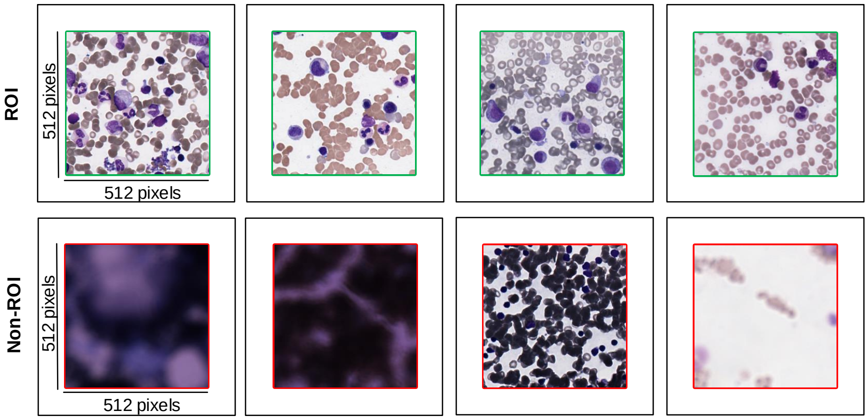

Figure S4: **Four Examples of applying the proposed cell detection and classification method to localize cellular objects (white blood cells) and other non-cellular objects and also classify them with the probability in the tiles.**

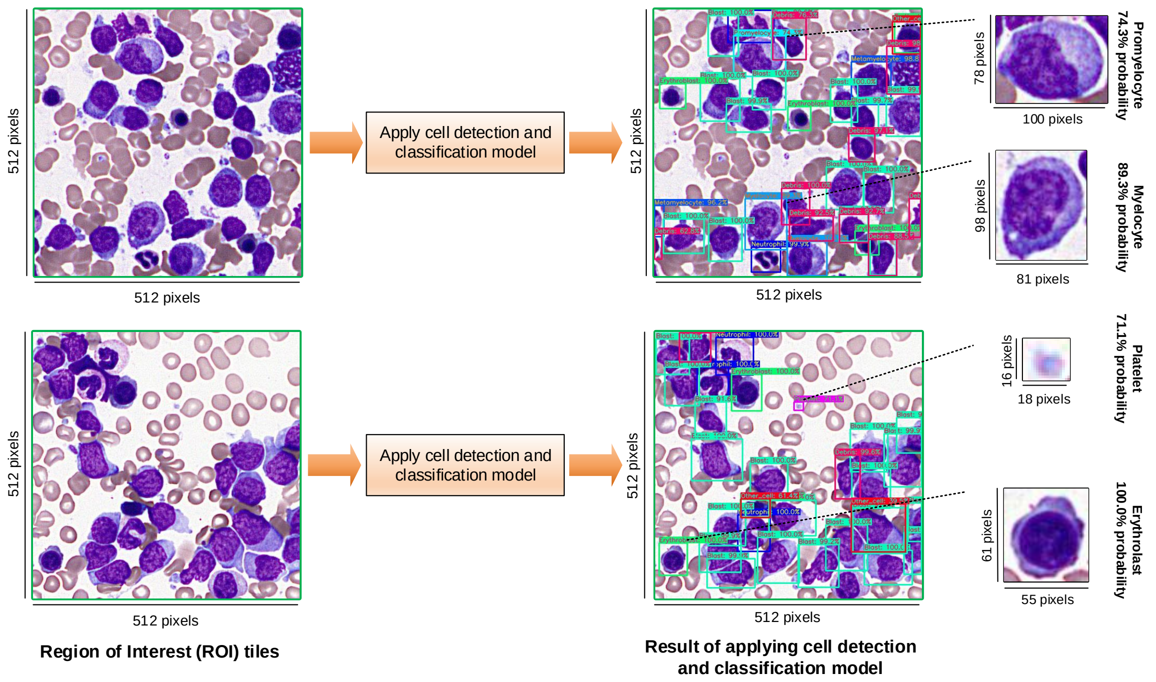

(a)

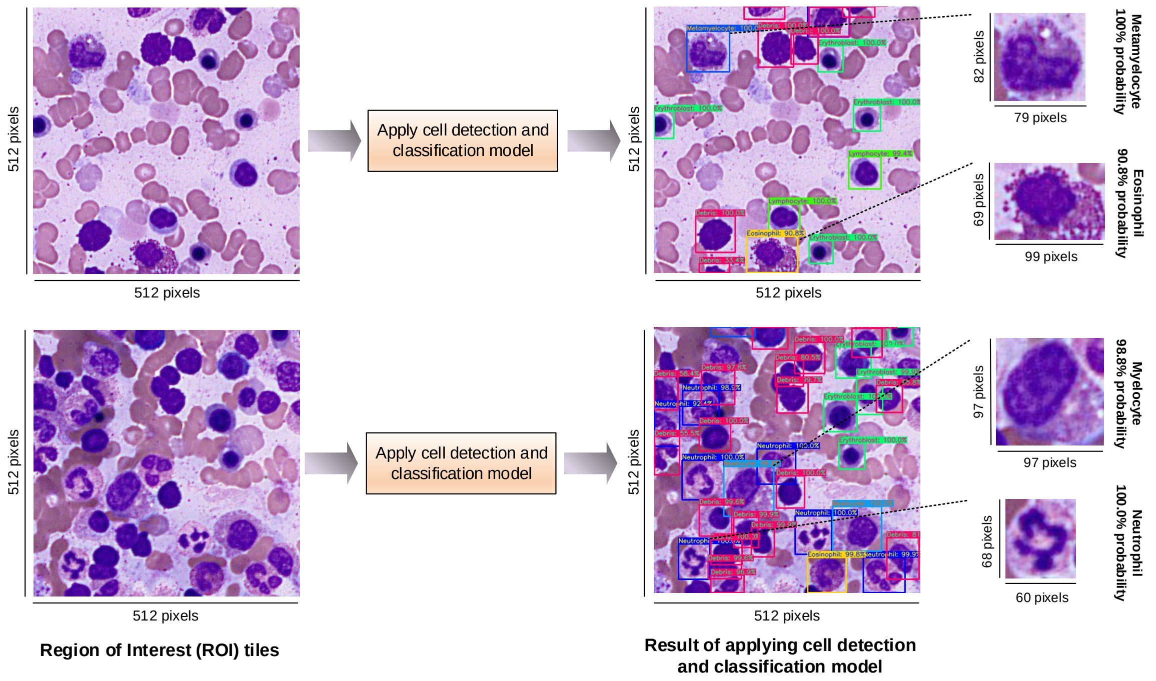

(b)

(a) Samples of applying the proposed cell detection and classification method on push preparation aspirate specimen. (b) Samples of applying the proposed cell detection and classification method on crush (squash) preparation aspirate specimen.

Figure S5: **The cross-validation confusion matrix showing the performance of the YOLO cell detection and classification model applied on 16 different cytological and non-cytological object types. Each value represents the percentage of classification per object type across others. Rows indicate the ground-truth object class while columns display the object type predicted by the proposed model. The diagonal values indicate the true positive portion for each object type and the other values, outside of the diagonal, display the misclassification rates.**

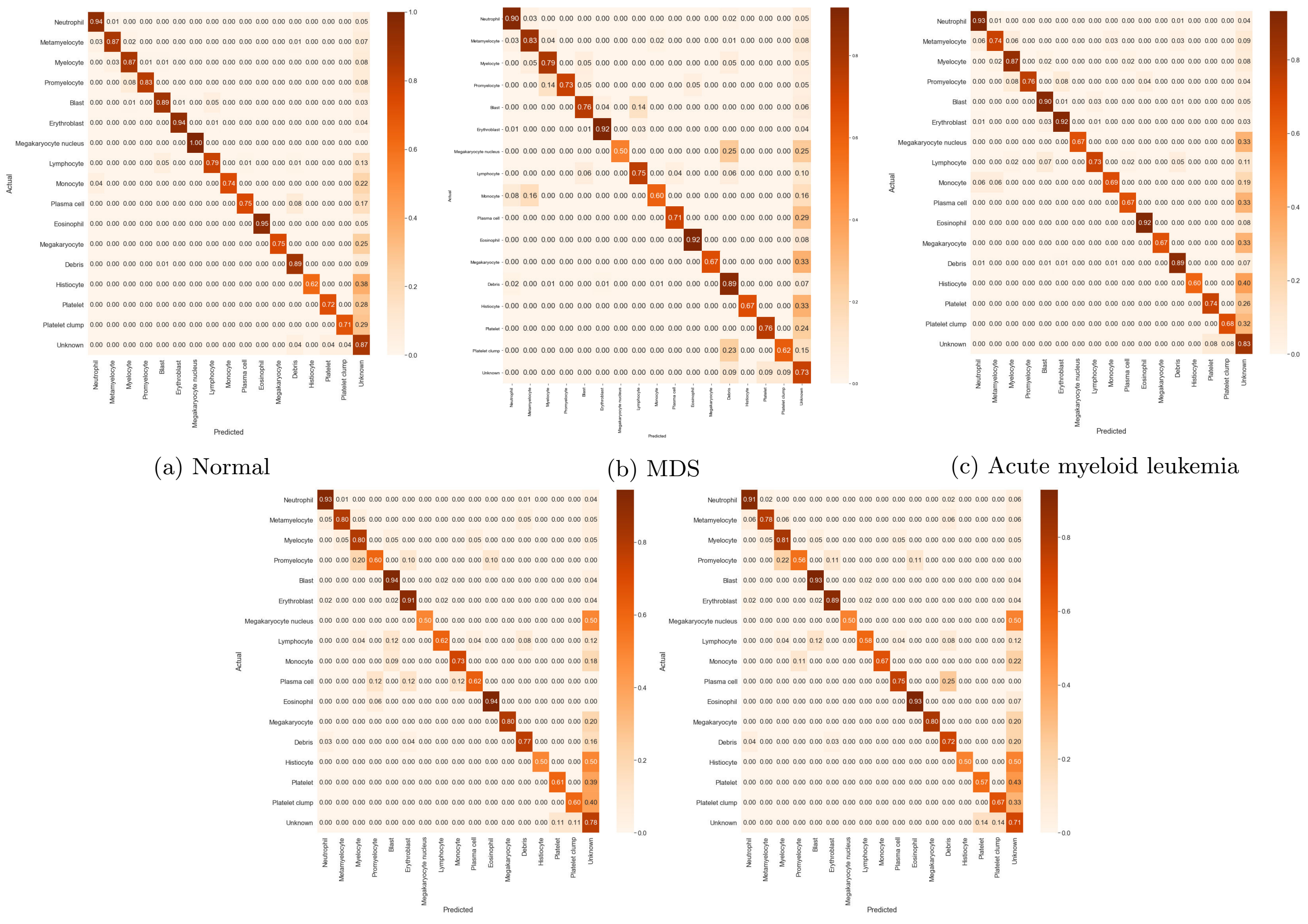

(a) Normal

(b) MDS

(c) Acute myeloid leukemia

(d) Plasma cell neoplasm

(e) Lymphoproliferative disorder

(a) The confusion matrix of applying the model on patient whole slide image (WSI) with normal diagnostic tags. (b) The confusion matrix of applying the model on patient whole slide image (WSI) with myelodysplastic syndrome (MDS) diagnostic tags. (c) The confusion matrix of applying the model on patient whole slide image (WSI) with acute myeloid leukemia diagnostic tags. (d) The confusion matrix of applying the model on patient whole slide image (WSI) with plasma cell neoplasm diagnostic tags. (e) The confusion matrix of applying the model on patient whole slide image (WSI) with lymphoproliferative disorder diagnostic tags.

Figure S6: **Two samples of the clinical validation.**

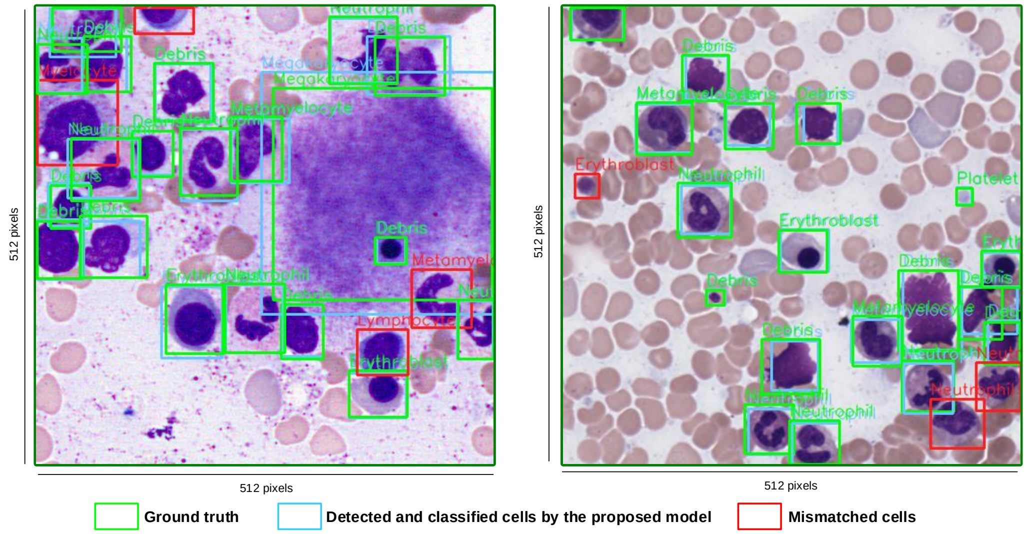

As shown, the result of applying the cell detection and classification model and the ground truth (the tiles checked with hematopathologists) viewed in single image to make it easier to investigate more for validation. Here, green bounding boxes and classes names indicate the ground truth, blue bounding boxes and classes names denote the result of cell detection and classification model which matched the ground truth, and red bounding boxes mark those detected objects that did not match the ground truth; the red class names, indicate the wrong prediction class name by the model.
